# Anti-Ro52 Antibodies and Interstitial Lung Disease in Connective Tissue Diseases Excluding Scleroderma

**DOI:** 10.5402/2012/415272

**Published:** 2012-03-27

**Authors:** João Pedro Ferreira, Isabel Almeida, António Marinho, Conceição Cerveira, Carlos Vasconcelos

**Affiliations:** ^1^Unidade de Imunologia Clínica, Hospital de Santo António, Centro Hospitalar do Porto, 4099-001 Porto, Portugal; ^2^Centro Hospitalar do Porto, Largo Prof. Abel Salazar, 4099-001 Porto, Portugal

## Abstract

*Introduction*. The presence of anti-Ro52 antibodies has been reported in a wide variety of autoimmune diseases, particularly in myositis, scleroderma, and autoimmune liver diseases. Clinical significance of anti-Ro52 antibodies remains controversial, and studies are lacking for clarifying the association of anti-Ro52 with interstitial lung disease (ILD) in connective tissue diseases (CTD). *Objectives*. To determine if anti-Ro52 antibodies are associated with ILD in CTD other than scleroderma. *Methods*. Single-center, retrospective study based on immunoblotting panel analysis and patients clinical records. *Results*. In our connective tissue disease cohort, 162 patients had immunoblotting panels with anti-Ro52 reactivity analysis, 41 (25,3%) had inclusion criteria. Among the 41 selected sera, 85.4% (*n* = 35) had anti-Ro52 reactivity. The prevalence of ILD in the positive anti-Ro52 antibodies was 71.4% (*n* = 25), and 16.7% (*n* = 1) in the negative anti-Ro52 group (*P* = 0.018). Overall sensitivity (96.2%), specificity (83.3%), positive (71.4%) and negative (83.3%) predictive values of anti-Ro52 antibodies to determine ILD in CTD is detailed in this study. *Conclusion*. Ro52 autoantibodies are associated with ILD in CTD excluding scleroderma. We suggest that the presence of anti-Ro52 reactivity in CTD should increase the clinician curiosity for the search of ILD.

## 1. Introduction

Antibodies to SSA antigen (Ro52/Ro60) were historically described as a marker for Sjögren syndrome and systemic lupus erythematosus [1]. However, recent publications [2, 3] have demonstrated that Ro52 and Ro60 (SSA) antigens consisted of two different proteins representing two distinct autoantibodies systems and have different clinical associations [4].

The Ro52 gene has been mapped to the end of the short arm of human chromosome 11 [5], and Ro52 antigen has recently been identified as a 52 kDa protein, belonging to the tripartite motif (TRIM) protein family [6]. So, anti-Ro52 antibodies can also be named as anti-TRIM21 antibodies.

The presence of anti-Ro52 antibodies has been reported in a wide variety of autoimmune diseases, particularly in myositis, scleroderma, and autoimmune liver diseases [7, 8]. These antibodies have also been associated with nonautoimmune diseases such as viral infections and neoplastic diseases [9]. Despite these associations, the clinical significance of anti-Ro52/TRIM21 antibodies remains controversial [10, 11].

As previously reported [7, 12], anti-Ro52 is the most common autoantibody detected in polymyositis with antisynthetase syndrome. Some studies report an association of anti-Ro52 antibodies with aggressive anti-tRNA synthetases syndrome [13, 14].

The main clinical reported data associate anti-Ro52 antibodies with interstitial lung disease (ILD) [7]. The reason why anti-Ro2 antibodies cause ILD is not established. It has been described that Ro52 targets many transcription factors to disregulate proinflammatory cytokine production belonging to the IL23-TH17 pathway and links to the development of tissue-specific inflammation and systemic autoimmunity [15]. Some authors find an association between the presence of anti-Ro52 antibodies and pulmonary infections. This association may be related to the gene location of Ro52 antigen on human chromosome 11. Also, the chromosome 11 (p15.5 segment) may harbor genes involved in the development and progression of lung cancer, and the Ro52 is a candidate tumor suppressor because of its function as a transcriptional regulator [15].

The paucity of information regarding our ability to predict pulmonary involvement in patients with connective tissue disease has led to the development of this study.

## 2. Objective

Our objective is to determine if anti-Ro52 antibodies are associated with interstitial lung disease (ILD) in connective tissue diseases (excluding scleroderma).

## 3. Methods

### 3.1. Studied Population

 Single center, retrospective study based on immunoblotting panels (EUROLINE) analysis and patients clinical records. All patients who had been screened for anti-Ro52 reactivity and were listed in the database of the immunology laboratory of our University Hospital in Porto between 1 January 2010 and 1 June 2011 were investigated.

 Autoimmune disease (AD) was defined when a patient displayed one of these: myositis with or without antisynthetase antibodies, systemic lupus erythematosus (SLE), rheumatoid arthritis (RA), Sjögren syndrome (SS), and mixed connective tissue disease (CTD). All in accordance with the international criteria for classification [16, 17]. Undifferentiated connective tissue disease (UCTD) was defined as an autoimmune disorder in which signs and symptoms are widely variable and evocative for connectivitis but not sufficiently evolved to fulfil any of the accepted classification criteria for the defined connective tissue diseases. Paraneoplastic syndrome was defined when a patient had a connective tissue manifestation of an established primary neoplasm.

### 3.2. Exclusion Criteria

Patients without connective tissue disease (CTD) diagnosis criteria as defined above, whose clinical records were insufficient, and patients without a pulmonary computed tomography (CT) scan were excluded.

Patients with systemic sclerosis (SSc) and autoimmune hepatitis were excluded. Concerning SSc, this study is under development by the same authors.

### 3.3. Definitions

 ILD was defined as alveolitis (ground glass changes) and/or fibrosis, determined by radiologist interpretation of high-resolution CT scan (HRCT). Whenever possible, these alterations were supported by nonspecific inflammatory changes (lymphocyte predominance) in bronchoalveolar lavage (BAL) results.

### 3.4. Data Analysis

 Data were registered in Excel, and SPSS software was used for statistical analysis.

 For proportion analysis, chi-square or Fisher tests were used. A significance (*P*) < 0,05 was defined.

 Complementary information in data analysis is mentioned along the text and tables.

## 4. Results

Of 162 immunoblotting panels analyzed for anti-Ro52 reactivity, 41 (25.3%) had inclusion criteria: 78% (*n* = 32) were women and 22% (*n* = 9) were men, mean (SD) age 50.5 (15.4) years.

We had to exclude 121 patients. The reasons were (1) no clear diagnosis (*n* = 24) of connective tissue disease (e.g., isolated Raynaud, arthralgia, dermatologic, and/or nonspecified pulmonary alterations), (2) inexistence of pulmonary HRCT scan (*n* = 53), and (3) patients from other centers (we had no access to clinical data of these patients) whose immunoblotting panels were processed in our center (*n* = 44).

 Among the 41 selected sera, 85.4% (*n* = 35) were positive for anti-Ro52 autoantibodies.

The gender distribution and mean age (SD) in the positive anti-Ro52 group were similar to the initial group (77.1% women; 50.3 (15) years, resp.).

 The prevalence of ILD in the positive anti-Ro52 antibodies was 71.4% (*n* = 25) and 16.7% (*n* = 1) in the negative anti-Ro52 group (*P* = 0.018).

Most patients had no respiratory symptoms described. Only the patients with antisynthetase syndrome (*n* = 5) had clear respiratory symptoms described and underwent BAL.

 Overall sensitivity (96.2%), specificity (83.3%), and positive (71.4%) and negative (83.3%) predictive values of anti-Ro52 antibodies for determining ILD in CTD are detailed in Table 1.

 The distribution of positive anti-Ro52 antibodies through CTD is shown in Table 2. No significant differences between CTD were found (*P* = 0.668).

 The distribution of ILD through CTD is detailed in Table 3. No significant differences between CTD were found (*P* = 0.996).

In absolute numbers, the majority of reported cases were associated with polymyositis/dermatomyositis (PM/DM), systemic lupus erythematosus (SLE), and mixed CTD. These diseases are analyzed separately below.

Concerning the 17 PM/DM cases, 76.5% (*n* = 13) had anti-Ro52 reactivity, and 64.7% (*n* = 11) had ILD. Of the 11 patients with ILD, 10 (90.0%) were anti-Ro52 reactive. No significant differences between groups were found (*P* = 0.09). In the 11 patient with ILD group, 5 (45.5%) had antisynthetase syndrome with anti-Jo1 positive autoantibody. All patients with anti-Jo1 antibodies (*n* = 5) were also anti-Ro52 positive. No significant differences between anti-Ro52 positive and negative groups were found (*P* = 0.208).

Analyzing the 9 SLE cases, 77.8% (*n* = 7) were anti-Ro52 positive, and 69.7% (*n* = 6) had ILD. All patients with ILD were anti-Ro52 positive (100%; *n* = 6). No significant differences between anti-Ro52 positive and negative groups were found (*P* = 0.083).

In the 8 mixed CTD group, 100% (*n* = 8) were anti-Ro52 positive, and 62.5% (*n* = 5) had ILD. In this particular case, no statistics were computed because anti-Ro52 is a constant.

## 5. Discussion

 As described in the introduction, anti-Ro52 antibodies have been detected in various autoimmune diseases and the main clinical reported data associate anti-Ro52 antibodies with ILD. On the other hand, some series do not consistently associate anti-Ro52 antibodies with autoimmune disease and find these autoantibodies weakly predictive of autoimmunity [18, 19].

Given the available data, we can describe the clinical significance of anti-Ro52 antibodies as *controversial*.

Our study demonstrates that anti-Ro52 antibodies are significantly associated with ILD in CTD and are very sensitive for ILD diagnosis. But they have less value for confirming and excluding ILD. A multicentric retrospective study [10] involving 155 patients whose sera was reactive to Ro52, looked at the clinical relevance of anti-Ro52 antibodies in autoimmune and nonautoimmune diseases. This study had a high prevalence of autoimmune diseases (73%). The prevalence of ILD associated with the presence of anti-Ro52 antibodies was 22% (34/155). These data are consistent with the data presented in our study.

 Whether anti-Ro52 antibodies could be a marker for ILD independent of the presence of CTD is a question we cannot answer in this small study. Further and larger studies must be designed to address this issue.

 Anti-Ro52 antibodies are present throughout CTD spectrum. When we analyze CTD separately, the differences between Ro52 positive and negative groups were not significant, maybe because absolute numbers were low and only Fischer tests could be used. Despite of these limitations we found a tendency towards significant differences in PM/DM (*P* = 0.09) and SLE (*P* = 0.083). Other studies did not find value for the positive or differential diagnosis of AD [19]. All patients (*n* = 5) with anti-tRNA synthetases syndrome were anti-Ro52 positive. No significant differences between groups were found. Once again low absolute numbers limited the statistical analysis. The presence of anti-Ro52 antibody could be associated with a poor prognosis or may precede the development of pulmonary infection in anti-tRNA synthetases syndrome. Close followup of those patients should be recommended [20].

Further studies should be addressed to clarify if Ro52 is significantly associated with ILD in each CTD separately, particularly the antisynthetase syndrome.

Our study has several limitations, the most important concerning the sample size and retrospective analysis. In our study, few patients had inclusion criteria (*n* = 41). The small sample size limits the power of this study. Given the heterogeneity of the sample, patients were followed by many practitioners of different areas of specialization. In addition, there was no standardization in follow-up or clinical registries. Consequently, we could not describe symptoms adequately nor assess the longer-term risk of developing ILD in patients with anti-Ro52 antibodies, and prospective monitoring is required to evaluate this risk. Finally, we had to exclude many patients (e.g., patients without pulmonary HRCT scan).

Despite the great limitations, we find some data addressed in this study an important matter of discussion.

## 6. Conclusion

 Ro52 autoantibodies are associated with ILD in CTD excluding scleroderma. In this clinical context, these auto-antibodies are very sensitive for ILD diagnosis. We suggest that the presence of anti-Ro52 reactivity in CTD should increase the clinician curiosity for the search of ILD.

## Figures and Tables

**Table 1:** Sensitivity (Ss), specificity (Sp), positive (PPV), and negative (NPV) predictive values of anti-Ro52 reactivity in determining pulmonary involvement in connective tissue diseases.

ILD	PARo52A	(CI _95%_)			
		Ss, %	Sp, %	PPV, %	NPV, %
*n* = 26	*n* = 35	96.2 (89–100)	83.3 (53–100)	71.4 (56–86)	83.3 (53–100)

ILD, interstitial lung disease; PARo52A, positive anti-Ro52 antibodies.

**Table 2:** Distribution of anti-Ro52 antibodies through connective tissue diseases.

	PM/DM *n* (%)	SLE *n* (%)	Sjögren *n* (%)	Mixed CTD *n* (%)	U CTD *n* (%)	P Neo *n* (%)	*P*
PARo52A	13 (76.5)	7 (77.8)	2 (100)	8 (100)	3 (100)	2 (100)	0.668*

PARo52A, positive anti-Ro52 antibodies; PM/DM, polymyositis dermatomyositis; SLE, systemic lupus erythematosus; CTD, connective tissue diseases; U, undifferentiated; P Neo, paraneoplastic.

*Fischer test.

**Table 3:** Distribution of interstitial lung disease (ILD) through connective tissue diseases (CTD).

	PM/DM *n* (%)	SLE *n* (%)	Sjögren *n* (%)	Mixed CTD *n* (%)	U CTD *n* (%)	P Neo *n* (%)	*P*
ILD	11 (64.7)	6 (69.7)	1 (50)	5 (62.5)	2 (66.7)	1 (50)	0.996*

PM/DM, polymyositis dermatomyositis; SLE, systemic lupus erythematosus; CTD, connective tissue diseases; U, undifferentiated; P Neo, paraneoplastic.

*Fischer test.
